# Clinical, hormonal and chromosomal analysis of undervirilized male/46XY DSD – a 3years experience of national institute of child health

**DOI:** 10.1186/1687-9856-2013-S1-P184

**Published:** 2013-10-03

**Authors:** Irum Atta, Saira Lone, Yasir Naqi Khan, Mohsina Ibrahim, Jamal Raza

**Affiliations:** 1 National Institute of Child Health, Karachi, Pakistan

## Objective

To do the clinical, hormonal and chromosomal analysis in undervirilized male / 46XY DSD. To make a Presumptive diagnosis on the basis of clinical, chromosomal and hormonal assessment.

## Methodology

This study was conducted in National Institute of Child Health at Department of Pediatrics, Division of Endocrinology from January 2008 to December 2010. A Total of 127 Patient under age of 14 years with ambiguity, micropenis, hypospadias, cryptorchism and delayed puberty were selected and studied.USG Pelvis, HCG Stimulation test and Chromosomal analysis were carried out in all patients. Two types of HCG stimulation test were performed. Short HCG was done in children ten and less than ten years of age. Prolong HCG was performed in children more than ten years of age. Laproscopy and biopsy were carried out in patients who had mullerian duct structure on USG and also in patients with no gonads. FISH analysis was done in patients who were 46XX karyotype with testes.

## Result

Total no. of patients were 127. 43% presented with hypospadias, 17% with ambiguity, 20% with cryptorchism, 13% with micropenis and 5 % with delayed puberty.HCG stimulation showed high response (pre and post- testosterone) in 29%, flat in 28%, partial in 27% and normal in 16% of patients. On chromosomal analysis 123 (97%) patients were turned out to be 46XY, 3(2%) patients were 46XX and 1(1%) patient was 46XXY. FISH analysis performed in 46 XX patients showed Y translocation in one patient. 8(6%) 46 XY DSD patients had both wolffian and mullerian duct structure on ultrasonography. Laproscopy and biopsy performed in 4(3%) patients and proved ovotesticular DSD on histopathology. Laproscopy was also done in 2(1.5%) 46 XY patients with no gonads on ultrasonography and diagnosed as a case of testicular regression syndrome on per-operative findings. The diagnosis of Gonadal dysgenesis considered in patients who have partial testosterone response, androgen insensitivity in high testosterone response and testicular biosynthetic defect in flat pre and post testosterone response to HCG.

## Conclusion

Phenotypic presentation of 46XY DSD depends on the underlying defects. Defect in androgen action on the target tissues or production of active metabolite share common morphological features. Molecular study may help in differentiating these abnormalities and to make a final diagnosis.

